# ASO Author Reflections: SPIO, a New Sentinel Node Tracer with Interesting Future Clinical Applications

**DOI:** 10.1245/s10434-019-07520-7

**Published:** 2019-06-17

**Authors:** Fredrik Wärnberg, Andreas Karakatsanis

**Affiliations:** Department of Surgical Sciences, Uppsala University, Uppsala, Sweden

## Past

Superparamagnetic iron oxide nanoparticles (SPIO) is a new tracer for sentinel node (SN) detection with comparable results to dual technique, Technetium^99^ ± blue dye.^1^ SPIO has not been associated with allergic reactions, and nuclear medicine facilities are not required. Concerns have however been raised regarding artifacts on magnetic resonance imaging (MRI) and brownish skin staining.

At Uppsala University Hospital, SPIO has routinely been used for 3 years. It has simplified planning for surgery, partly due to the possibility of injecting SPIO up to 4 weeks before surgery.^2^ To avoid skin staining, our injection technique was modified and SPIO is now injected close to the tumor instead of behind the areola as in earlier studies. Blue dye also results in skin staining, but little is reported on the cosmetic outcome. Here, women were asked^3^ about their experience of SPIO staining in relation to different injection techniques, and the SN detection rate was recorded.

## Present

SPIO was injected in different ways and at different time points. Peritumoral, in comparison with retroareolar injection, resulted in less skin staining with comparable sentinel node detection rates. Most women did not consider skin staining a cosmetic problem. Three years after peritumoral injection, 9.4% had a residual stain, but it was regarded as “no” or “a minor cosmetic problem” by 88% of the women. The staining faded and the size diminished successively over time. The volume of SPIO used was 2 ml, diluted in 3 ml NaCl or local anesthetic. We expect that staining could be decreased further by using smaller volumes of nondiluted SPIO, as we have promising results in an ongoing study.

Using different injection sites resulted in similar SN detection rates. However, injecting the SPIO before the day of surgery increased the SN detection rate by about 4% (98.0% vs. 94.2%, *p* = 0.06), with 0.3 more SNs retrieved. As we usually operate within 4 weeks, SPIO can be injected at the outpatient visit, when planning surgery.

## Future

SPIO has recently been Food and Drug Administration (FDA) approved, but only for mastectomy patients. However, we see several new possibilities. In ductal carcinoma in situ (DCIS) patients, SPIO is injected at primary surgery but SN biopsy (SNB) is not performed. In those upgraded to invasive cancer, SNB is performed at a second operation. This reduces unnecessary SNBs and saves money.^4^ The concept can be applied to prophylactic mastectomies.

The combination of SPIO and a magnetic seed for SN detection and tumor localization can simplify logistics further. SPIO and the seed are injected up to 4 weeks before surgery by the radiologist.^5^

Long-term MRI effects need to be studied, but by using a small peritumoral dose, most SPIO is removed at surgery and preliminary data show that artifacts are minimized.

Lastly, a SPIO-loaded SN can preoperatively be identified by magnetometer-guided ultrasound. This could allow it to be clipped prior to commencing neoadjuvant chemotherapy. Ultimately, magnetometer-guided biopsies of the SN could supersede surgical SNB.
